# Early Visual Saliency Based on Isolated Optimal Features

**DOI:** 10.3389/fnins.2021.645743

**Published:** 2021-04-30

**Authors:** Serena Castellotti, Anna Montagnini, Maria Michela Del Viva

**Affiliations:** ^1^Department of Neurofarba, University of Florence, Florence, Italy; ^2^Institut de Neurosciences de la Timone (UMR 7289), CNRS and Aix-Marseille Université, Marseille, France

**Keywords:** fast vision, information maximization, visual saliency, information-optimal local features, saccadic orientation

## Abstract

Under fast viewing conditions, the visual system extracts salient and simplified representations of complex visual scenes. Saccadic eye movements optimize such visual analysis through the dynamic sampling of the most informative and salient regions in the scene. However, a general definition of saliency, as well as its role for natural active vision, is still a matter for discussion. Following the general idea that visual saliency may be based on the amount of local information, a recent constrained maximum-entropy model of early vision, applied to natural images, extracts a set of local optimal information-carriers, as candidate salient features. These *optimal* features proved to be more informative than others in fast vision, when embedded in simplified sketches of natural images. In the present study, for the first time, these features were presented in isolation, to investigate whether they can be visually more salient than other *non-optimal* features, even in the absence of any meaningful global arrangement (contour, line, etc.). In four psychophysics experiments, fast discriminability of a compound of *optimal* features (target) in comparison with a similar compound of *non-optimal* features (distractor) was measured as a function of their number and contrast. Results showed that the saliency predictions from the constrained maximum-entropy model are well verified in the data, even when the *optimal* features are presented in smaller numbers or at lower contrast. In the eye movements experiment, the target and the distractor compounds were presented in the periphery at different angles. Participants were asked to perform a simple choice-saccade task. Results showed that saccades can select informative *optimal* features spatially interleaved with *non-optimal* features even at the shortest latencies. Saccades’ choice accuracy and landing position precision improved with SNR. In conclusion, the *optimal* features predicted by the reference model, turn out to be more salient than others, despite the lack of any clues coming from a global meaningful structure, suggesting that they get preferential treatment during fast image analysis. Also, peripheral fast visual processing of these informative local features is able to guide gaze orientation. We speculate that active vision is efficiently adapted to maximize information in natural visual scenes.

## Introduction

The visual system needs to analyze the visual scene efficiently in a short time—in the order of 10 ms, as fast image recognition is crucial for survival (Hare, 1973). A huge amount of information from the external world is potentially available, at any moment, to the visual system, thus the latter needs to quickly extract the most relevant elements to allow for an efficient adaptive behavior. A considerable amount of energy is indeed required to create an accurate representation of the visual scene in the shortest possible time (Attwell and Laughlin, 2001; Lennie, 2003; Echeverri, 2006). For this reason, the visual system is likely to operate a strong data reduction at an early stage of processing (Attneave, 1954; Barlow, 1961; Olshausen and Field, 1996), by creating a compact summary of the *relevant* features (Marr, 1982; Morgan, 2011).

While the existence of this early visual summary is rarely put into question, the principles driving the saliency of features, and the relative weight of local (Li, 2002; Zhang et al., 2012) and global cues in this process (Oliva and Schyns, 1997; Itti et al., 1998; Oliva, 2005), are still subject to intense debate. The saliency of a visual stimulus depends on several physical properties (typically luminance, color, orientation of isoluminant contours—edges) and it scales with the degree of dissimilarity of each property (e.g., luminance) with regard to the statistics of that property in the surround (e.g., the stimulus luminance vs. the background luminance, or the stimulus orientation compared to the orientation of the neighboring elements—see for instance Treisman, 1985; Nothdurft, 1993a, b). However, a stimulus’ saliency can often also be appreciated with isolated stimuli. Furthermore, the saliency related to each individual visual property of a single stimulus is typically combined into a global percept of stimulus saliency and different stimuli, defined by different conspicuous properties (e.g., a red square among green square and a tilted line among horizontal lines) can be compared and eventually empirically matched in terms of saliency (Nothdurft, 2000).

Several models have been proposed to quantitatively estimate the two-dimensional saliency distribution in a visual scene (the bottom-up saliency map). When considering more ecological conditions for vision, like during visual search with complex natural scenes, estimating the saliency of each part of the scene becomes much more difficult. Higher-level factors, such as object segmentation, semantic processing, and behavioral goals, do actually contribute, together with the physical properties, to define the relative conspicuity of the scene’s regions (for a review, see Fecteau and Munoz, 2006; Itti and Borji, 2013). Models of eye guidance have tried to predict where people fixate in visual scenes and to relate these locations to visual saliency. Some studies have suggested that eye movements are mainly driven by regions with maximal feature contrast (Itti and Koch, 2000, 2001; Itti and Baldi, 2009; Garcia-Diaz et al., 2012). Concurrently, in presence of multiple features, objects, and information cues, the pattern of ocular fixations in a complex natural scene is often used as the operational definition of the saliency map of the scene (Itti and Borji, 2013). Finally, the specific task at hand does also play an important role and for instance, it has been shown that eye movements statistics in humans are consistent with an optimal search strategy that gather maximal information across the scene to successfully achieve the task (Bruce and Tsotsos, 2005; Najemnik and Geisler, 2005, 2008; Garcia-Diaz et al., 2012).

**FIGURE 1:**
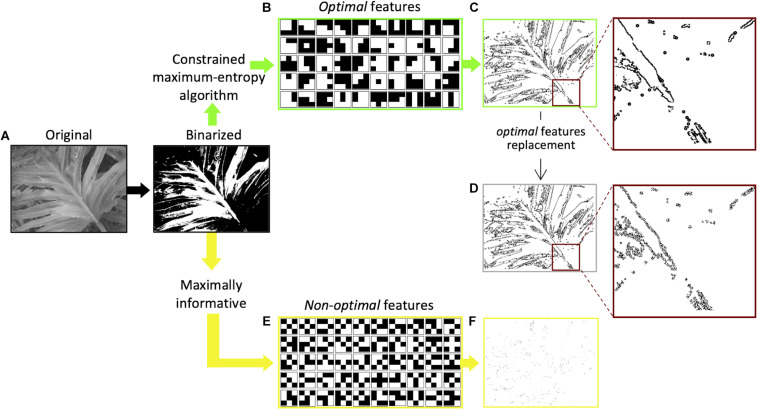
Extraction of *optimal* and *non-optimal* features and examples of sketches. **(A)** Original versions of natural images (Olmos and Kingdom, 2004) were first digitized into black and white. **(B)** Set of 50 3 × 3-pixels *optimal* features extracted by applying to black and white images the constrained maximum entropy model (Del Viva et al., 2013). **(C)** Sketch obtained by filtering the image with features in **(B)**. All the *optimal* features are positioned along image contours, as shown in the inset. **(D)** Sketch obtained by replacing in **(C)** the *optimal* features with *non-optimal* features (Del Viva et al., 2016). The spatial structure of the features along the contours does not correspond to the orientation of the contour, as shown in the inset. **(E)** A set of 50 3 × 3-pixels *non-optimal* features with the lowest probability in the statistical distribution of all possible 3 × 3-pixel black and white features. **(F)** Sketch obtained by filtering the digitized image with the 50 features in **(E)**.

The idea that the saliency of visual features is based on the amount of information (Shannon, 1948) they carry about the visual scene has been more recently proposed by Del Viva and colleagues (Del Viva et al., 2013). According to their model, in order to compress information and provide a saliency map of the visual scene, the visual system, at an early stage, acts as a filter that selects only a very limited number of visual features for further processing stages. The features selected are those that produce in output the largest amount of entropy, allowed by the given computing limitations—bandwidth and storage occupancy—of this early stage filter (*constrained maximum-entropy* model). Adopting the principle of maximum entropy as a measure of optimization, together with the imposed strict limitations to the computing resources of the system, allows to completely determine the choice of the features from the statistical distribution of the input data. The authors propose that only these *optimal* features, that are optimal carriers of information, are considered to be *salient* in fast vision. For economic reasons, and because the intent was to target early vision structures that have small receptive fields, the model was implemented by using very small features. Interestingly, the structure of the *optimal* features obtained by applying this constrained maximum-entropy model to a set of natural images (Olmos and Kingdom, 2004; Figure 1A), closely resembles the spatial structure of the well-known bar and edge-like receptive fields (Hubel and Wiesel, 1965) found in primary visual cortices (Figure 1B). This similarity implies that these specific visual receptive fields represent the optimal way to transmit information in early vision. On the other hand, features that do not fulfill the constrained maximum-entropy optimization criterion (*non-optimal*) happen to have either a uniform luminance structure (features with large bandwidth occupation) or a “noisy” alternation of black and white pixels (features with large storage occupation; see Figure 1E; Del Viva et al., 2013). Sketches, obtained by retaining only *optimal* features in black and white renditions of natural images (Figure 1C), and presented very briefly to human observers to ensure probing the early stages of visual analysis (Thorpe et al., 1996), allow very accurate discrimination. The discrimination of these sketches is comparable to that of their gray-scale original versions, although retaining only a small fraction of the original information (about 5–10%) (Del Viva et al., 2013). This shows that in fast vision it is sufficient to see very few details to discriminate images, provided that these few features are “the right ones.” In the sketches, *optimal* features turn out to be arranged along objects’ contours (edges and lines) rather than being scattered throughout the image, and the spatial structure of the features belonging to a particular contour corresponds to the nature and orientation of the contour (Figure 1C). That is, a vertical contour is composed of small local vertical edges (or lines). These authors showed also that, if *non-optimal* features are instead retained in the same black and white images, for example those with the lowest probability of occurrence (Figure 1E), the corresponding sketches are unrecognizable (Figure 1F). When using the latter, human discrimination performance drops dramatically (Del Viva et al., 2013).

The discrimination power provided by sketches based on *optimal* features could be due either to the specific local features used in the sketch or to their global spatial arrangement in the images. The contribution of individual local *optimal* features, located along with objects’ contours (global features), has been studied by replacing them with other features that are *non-optimal* carriers of information, keeping their localization along the contours unchanged (Figure 1D). That is, according to the example provided above, small local vertical edges (lines) in a vertical contour were replaced with different *non-optimal* local features, for example, the ones in Figure 1E. The disruption of these *optimal* local cues causes a decrease of image recognizability, despite its global structure being preserved (Del Viva et al., 2016).

Here we ask whether the *optimal* features identified in past experiments (Del Viva et al., 2013) are perceived as salient even when presented in isolation, outside the context of the global image structure to which they belong. We address this question through saliency discrimination between *optimal* and *non-optimal* features, by explicitly asking to the participants to choose the stimulus which stands out or grabs automatically their attention, through either a hand-button press, or a saccadic orienting response. This way of measuring saliency is not based on an automatic response, unlike in the majority of studies (e.g., Zhaoping and May, 2007; Donk and van Zoest, 2008), but it requires an explicit behavioral choice, as previously used, for example when preference does not depend on the intensity of a single low-level property of the stimulus—e.g., contrast, luminance, color (e.g., Nothdurft, 1993a; Nothdurft, 2000). This is the case of our stimuli, that do not differ for the low-level properties usually defining visual saliency, but for the internal spatial arrangement of black-and-white pixels (Figures 1B,E). These differences derive from a process of constrained-entropy maximization of the statistics of visual scenes, required by early input data reduction (Del Viva et al., 2013). Thus, the saliency preference for isolated *optimal* feature, even though asked explicitly, is not obvious.

Specifically, we conducted four psychophysics and one eye-movement experiment to determine the degree of saliency given by *optimal* features, compared to *non-optimal* features. In Experiment 1, to assess the minimal number of *optimal* features able to trigger a saliency discrimination, the preference for *optimal* vs. *non-optimal* features was measured as a function of their number. Experiment 2 was designed to assess how many *optimal* features surrounded by a group of *non-optimal* features (“signal-to-noise ratio,” SNR) are necessary to consider them more salient. This is a more ecological condition than that in Experiment 1 because in natural images *optimal* features (edges and lines) are always surrounded by others that do not define object contours and are therefore considered as noise, according to our model. Visual saliency is strongly dependent on luminance contrast (Treisman, 1985), whose analysis involves early visual processing starting from the retina. It is therefore particularly important to study its effect in determining saliency in fast vision. We studied the effect of contrast in Experiments 3 and 4. In Experiment 3, the preference for *optimal* vs. *non-optimal* features was measured as a function of the contrast of both, to measure the lowest contrast needed to still choose the *optimal* features as the more salient. In Experiment 4, the preference for the *optimal* features was measured as a function of their contrast relative to *non-optimal* features, to measure the minimal contrast *optimal* features must have to be considered as salient as *non-optimal* features. We can consider this value as the contrast equivalent to the saliency given by the spatial structure of *optimal* features. Finally, the preference for *optimal* features as a function of their number relative to *non-optimal* features (SNR), was also measured with saccadic eye movements. In this work, eye movements are not used as an operational definition of saliency (e.g., Itti and Borji, 2013), but as an alternative modality to the psychophysics response. We argue that the dynamic and metric properties of gaze-orienting responses might provide additional insight on the saliency-based capture exerted by optimal features.

## Materials and Methods

### Psychophysics Experiments

#### Observers

The condition with one feature in Experiment 1 was tested on 20 observers (13 women, mean age = 27 ± 2 years). Five other different observers (3 women, mean age = 23 ± 3 years) participated in the other conditions of Experiment 1, and Experiments 2, 3, and 4. All observers had normal or corrected to normal visual acuity and no history of visual or neurological disorders. Observers were unaware of the aim of the experiments (except for one author, in all experiments) and gave written informed consent before the experiments. All experimental procedures were approved by the local ethics committee (Comitato Etico Pediatrico Regionale—Azienda Ospedaliero-Universitaria Meyer—Firenze FI) and were compliant with the Declaration of Helsinki.

#### Apparatus and Set-Up

All stimuli were programmed on an ACER computer running Windows 7 with Matlab 2016b, using the Psychophysics Toolbox extensions (Brainard, 1997; Pelli, 1997; Kleiner et al., 2007), and displayed on a gamma-corrected CRT Silicon Graphics monitor with 1,280 × 960 pixels resolution at 120 Hz refresh rate. The whole display (38.5 × 29.5 cm) subtended 38.5 × 29.5° of visual angle at a viewing distance of 57 cm. All experiments were carried out in a dark room, with no lighting other than the display screen. *Ad hoc* software in Mathematica (Wolfram Inc.) was used for the extraction of stimuli, curve fitting, and statistical analysis. Participants’ manual responses were provided on a standard Dell keyboard.

#### Stimuli

Stimuli were two compounds of a certain number of small features, subtending 1.5 deg of visual angle at 57 cm distance (1.56 × 1.56 cm) and located horizontally at 3 deg eccentricity, right and left of the center of the screen. Each compound comprised several 3 × 3 pixels features, subtending 0.12 deg at 57 cm distance (0.12 × 0.12 cm) each. They were randomly selected with replacement (at each trial) from a set of 50 black and white *optimal* features selected according to the constrained maximum-entropy model, already used to build the sketches in previous experiments (Del Viva et al., 2013), and from a set of 50 black and white *non-optimal* features, with the lowest probability of occurrence in the statistical distribution of all possible 3 × 3 pixel black and white features (Figures 1B,E). The latter also were a subset of the *non-optimal* features, already used to build the sketches in previous experiments, specifically those fulfilling a maximum entropy but not a constrained-maximum entropy criterion (Del Viva et al., 2013). We chose these *non-optimal* features as a control for saliency because the difference between *optimal* and *non-optimal* features is given only by their internal black-and-white pixel arrangement, and they do not differ, on average, in luminance. *Non-optimal* features contain on average more of the higher spatial frequencies but there is a significant overlap of spatial frequency content between *optimal* and *non-optimal* features (Del Viva et al., 2013). More importantly, the spatial frequency spectrum of all our features lies entirely above the frequency of maximum human sensitivity. Our range of spatial frequencies is between 9 and 27 cycles/deg, while the maximum sensitivity lies at about 7 cycles/deg in our illumination conditions (Lamming, 1991). The positions of the features within each compound were assigned randomly at each trial and were set such that the distance between neighboring features had to be about 3 pixels in each direction. Random selection and random position of features in the stimulus ensured that saliency was provided only by individual features rather than by their global arrangement. The left/right position of each compound was also varied randomly from trial to trial. Luminance white: 35 cd/m^2^; luminance black: 1 cd/m^2^; luminance gray background: 12 cd/m^2^.

#### Procedure

**FIGURE 2:**
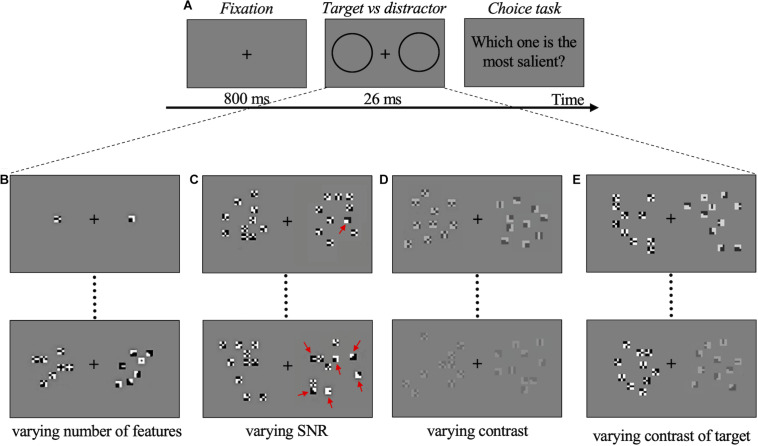
Psychophysics experiments procedure and conditions. **(A)** Schematic representation of one trial. During stimulus presentation two compounds, target (deemed salient), and distractor (not salient), were presented randomly left/right. The two black circles (not visible in the real display) represent the location of target and distractor, shown below in **(B–E)** for each experimental condition. In **(B–E**), the target is always on the right. **(B)** Examples of stimuli for Experiment 1. Upper panel: target with 1 optimal feature vs. distractor with 1 non-optimal feature. Lower panel: target with 7 optimal features vs. distractor with 7 non-optimal features. **(C)** Examples of stimuli for Experiment 2. Upper panel: target with 1 optimal feature plus 9 non-optimal features (SNR = 10%) vs. distractor with 10 non-optimal features. Lower panel: target with 6 optimal features plus 4 non-optimal features (SNR = 60%) vs. distractor with 10 non-optimal features. Red arrows indicate optimal features. **(D)** Examples of stimuli for Experiment 3. Upper panel: target with 10 optimal features vs. distractor with 10 non-optimal features (contrast of both = 50%). Lower panel: target with 10 optimal features vs. distractor with 10 non-optimal features (contrast of both = 20%). **(E)** Examples of stimuli for Experiment 4. Upper panel: target with 10 optimal features (contrast = 80%) vs. distractor with 10 non-optimal features (contrast = 100%). Lower panel: target with 10 optimal features (contrast = 65%) vs. distractor with 10 non-optimal features (contrast = 100%). Compounds and features are oversized for illustration purposes. The target-distractor compounds position in the trials is randomized.

In all experiments, participants were asked to choose which of the two compounds presented on each side of the screen was the most salient, in a 2AFC procedure. Participants were sitting in a dark room at 57 cm distance from the monitor. Each trial started with the presentation of a gray display for 800 ms, during which subjects were asked to fixate a cross in the center of the screen. The compound stimuli were then shown for 26 ms on a gray background. After the stimulus presentation, subjects indicated the more salient compound by pressing a computer key. There was no time limit for the response (Figure 2A). All data for each subject were collected in one single session of about 1 h, divided into four blocks (one block/experiment).

In Experiment 1 the preference for a compound of *optimal* features (target) with respect to a compound of *non-optimal* features (distractor) was measured as a function of the number of features presented. The luminance contrast was 100% in all trials. Considering these features are very small and are presented for a very short time, the minimal number of optimal features that triggers a consistent preference based on saliency becomes very important. For this reason, in a preliminary phase, a single feature was presented on each side, to check for the possible presence of an effect even in this limit condition. A total of 200 trials/observers were run. 20 observers participated just in this measurement. Then, five different observers completed the experiment to assess the number of features that produce maximal saliency discrimination. Three, five, seven, and ten features in each compound were presented to these five observers. Target and distractor always had the same number of features, varying from trial to trial according to a constant-stimuli procedure. A total of 1,200 trials per observer were run (Figure 2B).

In Experiment 2 the saliency-based preferential choice was measured as a function of the relative number of *optimal* vs. *non-optimal* features in the same compound. The target included a total of 10 *optimal* and *non-optimal* features in variable proportions (variable signal to noise ratio, SNR). The distractor included 10 *non-optimal* features. The luminance contrast (100%) and the total number of features in each compound (10) were kept constant in all trials. The SNR was either 0.1, or 0.4, or 0.6 or 1 (corresponding to 1, 4, 6, or 10 *optimal* features in the target compound), and this number was set randomly from trial to trial according to a constant stimuli procedure. A total of 1,200 trials per observer were run (Figure 2C).

In Experiment 3, the strength of the saliency-based preferential choice was measured as a function of the contrast of both *optimal* features (target compound) and *non-optimal* features (distractor compound). In this experiment, the number of features in the two compounds was kept constant at 10. Contrast for both target and distractor was set at 0.15, 0.2, 0.25, 0.3, 0.5 and the value was set randomly from trial to trial according to a constant stimuli procedure. Subjects were asked to press a computer key to indicate the more salient compound. A total of 500 trials per observer were run (Figure 2D).

In Experiment 4, the preference for *optimal* features (target compound) was measured as a function of their contrast relative to the contrast of *non-optimal* features (distractor compound). That is, in half of the trials, the contrast of the target was varied while the contrast of the distractor was kept constant at 100%. The contrast of the target for which the observers could not tell anymore which compound was more salient can be considered as 1-the contrast value equivalent to the saliency of our features. In the other half of the trials, the contrast of the target was kept constant at 100% while the contrast of the distractor was varied. These “catch trials” were used to avoid contrast cues that could bias the observers’ choice. All these trials were randomized. The number of features in the two compounds was the same (10) in all trials. Contrast values in the varying compound were 0.65, 0.70, 0.75, 0.80, 0.85, 0.90, 0.95, 1, set randomly from trial to trial according to a constant stimuli procedure. A total of 800 trials per observer were run (Figure 2E).

In Experiments 3 and 4, for every condition and subject, a 2-parameters (position and slope) Maximum Likelihood fit was performed off-line with data obtained in all sessions, based on an ERF (sigmoid) psychometric function. Psychometric functions run from 0.5 to 1 in Experiment 3 and thresholds were defined as the target contrast yielding 75% correct discrimination. In Experiment 4, psychometric functions run from 0 to 1, and thresholds were defined as the target contrast yielding 50% correct discrimination. The goodness of fit was determined from the difference in log-Likelihood between the fit, and an ideal fit describing all points exactly. This is used to obtain a *p*-value under the chi-square approximation (Wilks’ theorem).

### Eye Movements Experiment

#### Participants

Seven observers (3 women, mean age = 30.1 ± 8 years) participated in the eye movements task and the psychophysical control experiment. Five of them were completely naive to the goal of the experiment. All observers had normal or corrected to normal visual acuity and no history of visual or neurological disorders. All experimental procedures were approved by the local ethics committee (Comité d’éthique d’Aix-Marseille Université, ref: 2014-12-3-05) and were compliant with the Declaration of Helsinki.

#### Apparatus and Set-Up

All stimuli were programmed on a MacPro computer running OS 10.6.8 with Matlab 2016b, using the Psychophysics Toolbox (Brainard, 1997; Pelli, 1997; Kleiner et al., 2007), and the Eyelink Toolbox extensions (Cornelissen et al., 2002), and displayed on a Samsung SyncMaster 2,233 LED-monitor with 1,680 × 1,050 pixels resolution at 120 Hz refresh rate. The whole display (47.2 × 29.5 cm) subtended about 47 × 29° of visual angle at a viewing distance of 57.3 cm. All experiments were carried out in a dark room, with no lighting other than the display screen. Eye movements were recorded using an Eyelink 1,000 video-based eye tracker (sampling rate 1 kHz). The viewing was binocular, but only the right eye was tracked. A chin and forehead rest stabilized the head.

#### Stimuli

**FIGURE 3:**
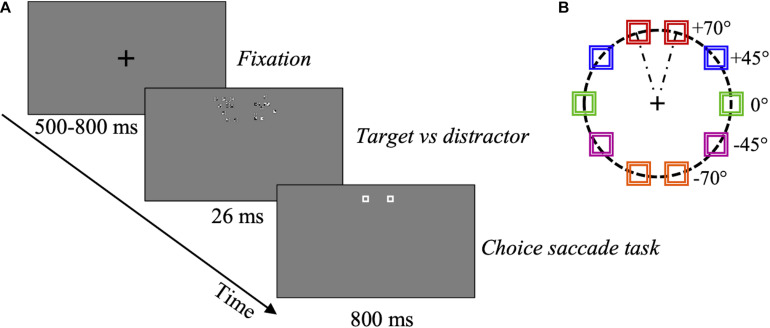
Eye movements experiment procedure. **(A)** Schematic representation of one trial. After a random-duration fixation period, one compound comprising a variable proportion (SNR) of optimal features (target), and a compound with non-optimal features (distractor), were presented to the right or left, at different angles, for 26 ms. Then, two placeholders were shown at the target and distractor locations for 800 ms. In this example, target SNR = 60%. For an enlargement showing the configuration of the actual compounds see Figure 2C. **(B)** Target and distractor compounds could be presented randomly at one of 5 different locations (0°, ± 45°, ± 70°) as defined in the text, illustrated by different colors, at 5 deg eccentricity. Red: angle + 70°; blue: angle + 45°; green: angle 0°; violet: angle –45°; orange: angle –70°.

Stimuli were two compounds of 10 features each. The target compound comprised a variable amount of *optimal* features (1, 4, 6, or 10) and *non-optimal* features, the distractor comprised only *non-optimal* features, analogously to the psychophysics Experiment 2. For the eye movements experiment, the target and distractor compound-pair could appear randomly at 5 different locations, with target and distractor arranged symmetrically with respect to the vertical meridian and their respective position (right or left) randomly switched across trials (Figure 3). If we consider the compound on the right-hand side, its position was defined by an angle of 0°, ± 45°, or ± 70° with respect to the horizontal midline (Figure 3B). In the following, we will refer to these angles to indicate the position of the compound pair. Angles were randomly alternated in the presentation sequence to maximally reduce motor preparation for the saccade and to assess possible spatial anisotropies of the *optimal* features-based saliency. Both compounds were displayed at a larger eccentricity (5°) than the one used in the psychophysical experiments (3°), in order to elicit goal-directed saccades, clearly aiming outside the perifoveal region (Figure 3B). To compensate for the larger eccentricity, all the stimuli were slightly larger than in Experiment 2. Compounds subtended 1.8 × 1.8 deg (nearly 1.8 × 1.8 cm) and individual features about 10 × 10 min of arc (0.17 × 0.17 cm) at 57.3 cm viewing distance. Each feature was defined by a 6 × 6 white and black pixels patch. Positions of features within each compound were randomly assigned at each trial, ensuring a distance of about 6 pixels in each direction between neighboring features. White pixels had a luminance of 82 cd/m^2^; black pixels: < 2 cd/m^2^; and the luminance of the gray background was about 42 cd/m^2^.

#### Procedure

After a fixation period of random duration between 500 and 800 ms, the target-distractor pair was presented for 26 ms (three frames). Then, two placeholders were displayed for 800 ms at the compound-pair location. The placeholders ensured that observers could program a relatively accurate visually guided saccade even once the compounds have disappeared. Observers were asked to move their gaze toward the location where they saw the “most salient stimulus,” in a 2AFC choice-saccade task (Figure 3A). To assess the rapid and possibly most automatic response to *optimal* features, only the first visually guided saccade was considered in each trial. 800 trials were collected for each subject.

Since experimental conditions are different from the psychophysics Experiment 2, as a control, we repeated the psychophysical measurements with these observers, stimuli, and setup. As in the psychophysics Experiment 2, the saliency-based preferential choice for the *optimal* features was measured as a function of their number relative to the total number of features (*optimal* and *non-optimal*, always equal to 10) in the same compound (SNR). In this control experiment, only the condition where the target and distractor were presented on the horizontal axis was tested and a total of 400 trials/observer were run.

#### Eye Movements Data Analysis

*Ad hoc* software in Matlab and Mathematica (Wolfram Inc.) was used for extraction of oculomotor parameters and statistical analysis. Recorded horizontal and vertical gaze positions were low-pass filtered using a Butterworth (acausal) filter of order 2 with a 30-Hz cutoff frequency and then numerically differentiated to obtain velocity measurements. We used an automatic conjoint acceleration and velocity threshold method to detect saccades (see for instance Damasse et al., 2018), and we visually inspected all oculomotor traces to exclude aberrant trials. We excluded from the analysis saccades with latencies below 140 ms, considered anticipatory and not guided by visual information in this type of choice-saccade tasks (e.g., Walker et al., 1997), and very late saccades, above 500 ms (less than 6% of the first detected saccades overall). Visual inspection of individual latency histograms confirmed that saccades with latency below 140 ms and above 500 ms did not belong to the principal mode of the distribution. When a small anticipatory saccade was detected (amplitude below 3 deg), the second saccade was used instead for the analysis (less than 2% of total). For each saccade, we estimated latency, amplitude, endpoint position, and the distance between the eye position endpoint and the center of the target (or distractor) compound. Saccades ending within 1.5 deg of either target or distractor were classified as “valid,” and, respectively, labeled “To-target” (*correct*) or “To-distractor” (*erroneous*). All the other saccades, landing farther than 1.5 deg from the compound, were considered as invalid, and labeled “Quasi-Target” or “Quasi-Distractor” when they brought the gaze in the same hemifield of the target or the distractor, respectively. The choice of the 1.5° distance criterion was motivated, on one hand, by the requirement that the validity-surrounds would not overlap between the two compounds in the 70° (uppermost) and −70° (lowermost position) conditions. On the other hand, this criterion distance is reasonable for a target-compound with a side of approximately the same size.

## Results

### Psychophysics Experiments

**FIGURE 4:**
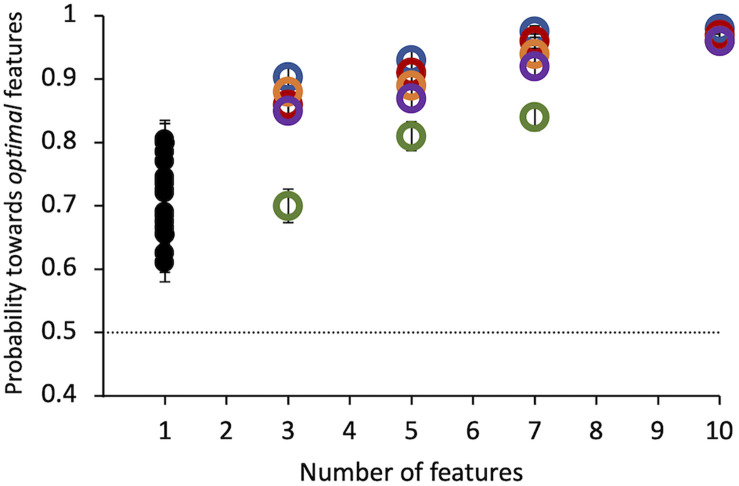
Experiment 1: Probability of selecting the target as a function of the number of features. Colored symbols represent data from 5 individual observers. Black symbols represent data from 20 different individual observers, tested in this condition only. Errors of individual dots are binomial standard deviations. The dashed line indicates the guessing level for this task (0.5 probability).

Results of Experiment 1 show that all observers found the target compound to be much more salient than the distractor. Even a single tiny *optimal* feature was chosen with probability = 0.71 ± 0.01 over its alternative by 20 observers (Figure 4). Probability of choosing the target as more salient increases with the number of features presented up to 10 features, where probability saturates for all subjects. This number was used in all the following experiments.

**FIGURE 5:**
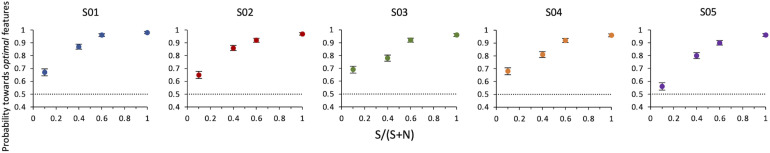
Experiment 2: Probability of selecting the target as a function of SNR. Data from individual observers. The dashed line indicates the guessing level for this task (0.5 probability). Target SNR could be 0.1, 0.4, 0.6, or 1 (corresponding to 1, 4, 6, or 10 optimal features in the target compound out of 10 total features).

Results of Experiment 2 show that even when *optimal* features are intermixed with *non-optimal* features in the same compound, observers still indicate this compound as more salient than the alternative. The probability increases with SNR. A compound with a single *optimal* feature surrounded by nine *non-optimal* features is sufficient to lead observers to consider this stimulus as more salient than the other with probability = 0.64 ± 0.02 (*z* = 6, *p* < 0.001) (Figure 5).

**FIGURE 6:**
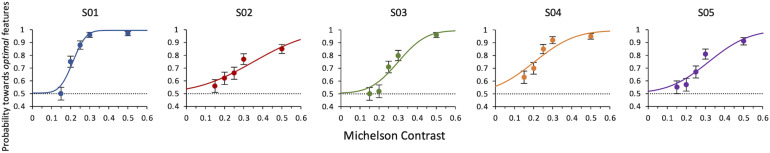
Experiment 3: Probability of selecting the target as a function of contrast of both target and distractor. Data from individual observers. The line is the ML best fit. Individual thresholds are given by contrast values corresponding to 75% level performance and are, respectively, 0.21 ± 0.007, 0.35 ± 0.88, 0.30 ± 0.02, 0.20 ± 0.02, 0.32 ± 0.02. The dashed line indicates the guessing level for this task (0.5 probability).

Results of Experiment 3 show that the lowest contrast needed to still choose the *optimal* features as the more salient is 0.23 ± 0.0006. This is the weighted average of the thresholds from maximum likelihood fits of individual data (Figure 6).

**FIGURE 7:**
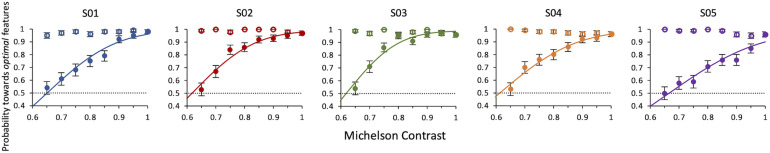
Experiment 4: Probability of selecting the target as a function of relative contrast of target or distractor. Data from individual observers. Filled symbols: the contrast of the target is varied. Open symbols: the contrast of the distractor is varied. The line is the ML best fit. Individual thresholds are given by contrast values corresponding to 50% level performance and are, respectively, 0.65 ± 0.01, 0.62 ± 0.006, 0.62 ± 0.01, 0.61 ± 0.01, 0.67 ± 0.01.

Results Experiment 4 show that when the contrast of *non-optimal* features is lowered, all observers always deemed the compound of *optimal* features (kept at 100% contrast) the most salient one. Conversely, when the contrast of *non-optimal* features was kept at 100% and that of *optimal* features was lowered, they still considered them as more salient, but with a decreasing probability as the contrast decreased. The average contrast value for which the contrast of *optimal* features balances the saliency of the *non-optimal* features is 0.63 **±** 0.004 (weighted average of individual thresholds) (Figure 7).

### Eye Movements Experiment

**FIGURE 8:**
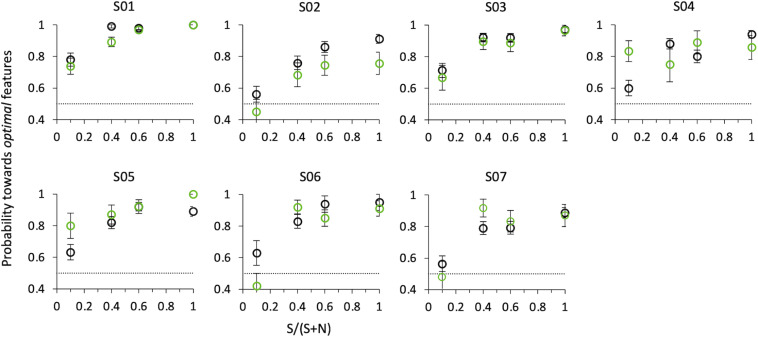
Probability of selecting the target as a function of SNR at angle 0°. Black circles: psychophysical results (button-press); green circles: eye movements results. Data from individual observers (weighted means with their errors). The dashed line indicates the guessing level for this task (0.5 probability).

Figure 8 shows probabilities for the first *correct* saccade and psychophysical choice of the same observers, as a function of the relative number of *optimal* vs. *non-optimal* features in the compound (SNR), when the target and distractor compounds were presented on the horizontal axis (0/180°). Both psychophysical and eye movements data confirm the results of Experiment 2, although with a smaller set of data and at a slightly larger eccentricity (5° instead of 3°).

That is, even when in the same compound *optimal* features are intermixed with *non-optimal* features, observers consider this compound as more salient than the other comprising only *non-optimal* features, and they do so with a probability that increases with SNR. A compound with just one *optimal* feature surrounded by nine *non-optimal* features is sufficient to lead observers to consider this stimulus as more salient than the other one with probability 0.65 ± 0.02 for psychophysics (*z* = 3.66, *p* < 0.001), and to orient the gaze toward it with probability 0.65 ± 0.03 for saccadic choice (*z* = 1.75, *p* < 0.05).

**FIGURE 9:**
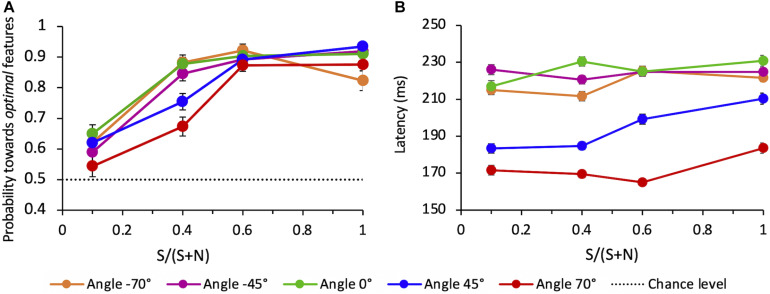
**(A)** Average probability of *correct* saccades as a function of SNR at different angles. Data are pooled across observers (weighted means with their errors). The dashed line indicates the guessing level for this task (0.5 probability). **(B)** Average latency of *correct* saccades as a function of SNR at different angles. Averages are taken over all correct trials (classified as “to-the-target”) and all observers (weighted means with their errors).

When all directions tested are considered (−70°, −45°, 0°, 45°, 70°), the average probability for the choice saccade to land in the vicinity of the target compound also increases with SNR (Figure 9A). The average performance depends on angles: compared to angle 0°, performance is lower for the upper quadrant, both for + 45° (*z* = −2.19, *p* < 0.05) and + 70° angles (*z* = −5.20, *p* < 0.001). The performance for the lower quadrant does not differ from 0°, either for −45° (*z* = 0.09, *p* > 0.5) and −70° angles (*z* = −1.20, *p* > 0.05).

We evaluated the mean latency of saccades that were correctly oriented toward the salient compound, as a function of the SNR and for each different angle of presentation. Figure 9B shows a strong difference of the saccadic latency across angles, with latencies being much shorter for eye movements directed toward the upper hemifield and in particular to the uppermost target-distractor compound location (angle + 70°). A mixed-effects linear regression analysis of mean saccade latency (with SNR, angle, and choice-accuracy–to-Target vs. to-Distractor saccades–as fixed-effects, and the same factors per subject as random-effects) revealed that only the angle but neither SNR nor choice-accuracy did significantly influence latency (mean regression slope = −0.46; standard error = 0.08; *t* = −5.63; *p* < 0.01).

**FIGURE 10:**
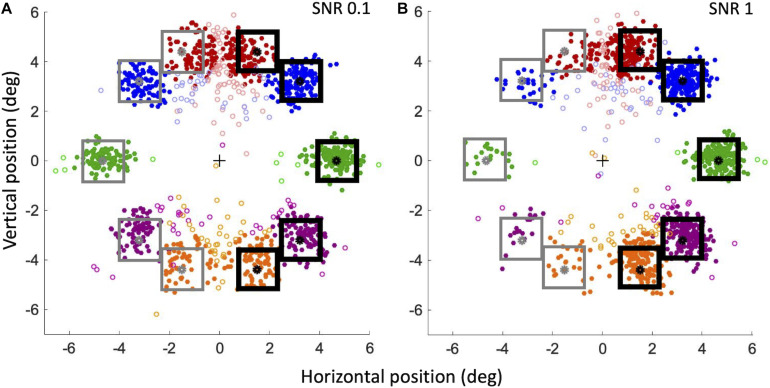
Landing positions of *correct* and *erroneous* saccades for SNR 0.1 **(A)** and SNR 1 **(B)**. For our convention, the center of the screen corresponds to a horizontal component of 0; the target compound (black squares) is always on the right hemifield, and the distractor compound is always on the left hemifield (gray squares). Filled circles: valid saccades (within 1.5°); empty circles: invalid saccades. Red circles: angle + 70°; blue circles: angle + 45°; green circles: angle 0°; violet circles: angle –45°; orange circles: angle –70°.

Figure 10 shows the landing point of all saccades of all subjects for the lowest (Figure 10A) and highest (Figure 10B) stimulus saliency conditions. To better visualize saccadic accuracy and precision, data have been flipped and pooled as though the target compounds were always on the right hemifield and the distractor compounds were always on the left hemifield. Saccades are categorized as *valid*, “to-Target” (*correct*), or “to-Distractor” (*erroneous*), when they land at a distance lower than 1.5°, respectively, from the target or distractor (filled circles in Figure 10).

To analyze the precision of landing positions for valid saccades, we calculated the absolute distance of landing position of *correct* and *erroneous* saccades from the compounds. At SNR 0.1, the mean absolute distance (± SEM) from the target compound of *correct* saccades was not significantly different from the distance of *erroneous* saccades from the distractor compound [respectively 0.74 ± 0.03° and 0.76 ± 0.03°; Paired Samples 1-tailed *t*-test, *t*_(__6)_ = 0.93, *p* > 0.05]. At SNR 1, the distance from the target of *correct* saccades was instead significantly smaller than the distance of *erroneous* saccades from the distractor [respectively, 0.59 ± 0.05° and 0.99° ± 0.09°; *t*_(__6)_ = 4.19, *p* < 0.01]. At SNR 1 *correct* saccades landed closer to the target than at SNR 0.1 [*t*_(__6)_ = −4.22, *p* < 0.01], whereas *erroneous* saccades at SNR 1 landed further away from the distractor than at SNR 0.1 [*t*_(__6)_ = 2.4, *p* < 0.05]. To investigate whether these differences could be explained by an “attraction” exerted by *optimal* features, we also analyzed the landing position along the horizontal axis, that is the presence of left-right biases in the saccades directions. *Landing errors* of *correct* and *erroneous* saccades were computed as the difference between the horizontal component of the estimated eye position at the end of the saccade and the position of the center of the nearby target or distractor compound. According to our convention (see caption of Figure 10), for to-Target saccades, a *landing error* compatible with zero corresponds to a saccade landing precisely on the target center; a negative *landing error* corresponds to a saccade landing closer to the screen vertical midline with respect to the target center (thus in the direction of the distractor on the horizontal axis), whereas a positive *landing error* corresponds to a saccade landing further away from the screen vertical midline (beyond the target on the horizontal axis). The opposite relation holds for to-Distractor saccades. At SNR = 0.1 (Figure 10A) the mean *landing error* for saccades to-Target (−0.09° ± 0.002) is significantly different from 0 [One Sample 2-tailed *t*-test, *t*_(__6__)_ = −4.80, *p* < 0.01]. The mean *landing error* for saccades to-Distractor (0.19° ± 0.07) is significantly different from 0 as well [*t*_(__6__)_ = 5.06, *p* < 0.1]. Thus, with low-saliency compounds, both to-Target, and to-Distractor saccades land nearer to the screen vertical midline. However, the absolute value of the *landing error* of to-Distractor saccades is larger than that of to-Target saccades [Paired Samples 1-tailed *t*-test, *t*_(__6__)_ = 3.004, *p* < 0.05], suggesting that to-Distractor saccades are less precise than to-Target saccades and that they tend to land shorter from the distractor and relatively closer to the salient compound on the opposite side. At SNR = 1 (Figure 10B) the mean *landing error* for to-Target saccades (−0.03° ± 0.1) is not significantly different from 0 [*t*_(__6__)_ = −0.67, *p* > 0.5], whereas the mean *landing error* for to-Distractor saccades (0.28° ± 0.1) is significantly different from 0 [*t*_(__6__)_ = 3.89, *p* < 0.01], again significantly greater than that of to-Target saccades [*t*_(__6__)_ = 3.63, *p* < 0.01]. Thus, when the target compound is very salient, to-Target saccades are precise and land very close to the compound center, whereas to-Distractor saccades are less precise and tend to fall short of the distractor, revealing a bias for the saccade landing position toward the salient compound. In addition, when SNR increases from 0.1 to 1, the *landing error* for saccades to-Distractor move further away from the distractor compound and relatively closer to the target compound [*t*_(__6__)_ = −2.39, *p* < 0.05], whereas to-Target saccades land closer and closer to the center of the target compound [*t*_(__6__)_ = −2.21, *p* < 0.05], indicating that the bias observed in the *landing errors* is affected by the feature saliency. When analyzed independently for different angles, the precision of valid to-Target saccades does also provide different results. The mean *landing error* (± SEM) for the 70° angle is quite large: −0.48° ± 0.05 at SNR = 0.1 and −0.46° ± 0.04 at SNR = 1. In contrast, saccades are much more precise at 0° angle, with a *landing error* compatible with 0° within our uncertainty (−0.03° ± 0.04 at SNR 0.1; 0.01° ± 0.04 at SNR 1).

To assess the attraction of optimal features independently on the criterion we choose for validity, we also analyzed the behavior of invalid saccades landing farther than 1.5° from either target (“Quasi-Target”) or distractor (“Quasi-Distractor”), for the two extreme SNR values, 0.1 and 1 (empty circles in Figure 10). First, in order to measure saccade accuracy, the ratios of “Quasi-Target”/“to-Target” and “Quasi-Distractor”/“to-Distractor” saccades were compared. When considering all saccades independently of the angle, the “Quasi-Distractor”/“to-Distractor” ratio is larger than “Quasi-Target”/“to-Target,” both for the lowest (Binomial test, 18% vs. 11%, *p* = 0.004) and highest (18% vs. 9%, *p* = 0.0004) SNR values. This result suggests that when saccades are directed on the side of the distractor, the probability to meet the 1.5° criterion from the goal is lower, compared to saccades directed on the side of the salient compound. When different angles are considered separately, the landing position at 0° is the most accurate, with a low ratio of “Quasi-Target”/“to-Target” (3% at SNR = 0.1 and 1% at SNR = 1), becoming progressively less accurate moving further away from 0° (see Table 1). Then the horizontal landing position with respect to the vertical midline of the screen of these invalid saccades was analyzed, to detect possible biases due to saliency. At SNR = 0.1 (Figure 10A), the absolute horizontal landing position of “Quasi-Target” and “Quasi-Distractor” saccades are statistically compatible [*t*_(__6__)_ = 0.32, *p* > 0.5]. In contrast, at SNR = 1 (Figure 10B) Quasi-Distractor saccades land away from their goal and closer to the center of the screen compared to Quasi-Target saccades [*t*_(__6__)_ = 2.06, *p* < 0.05]. In addition, when SNR increases from 0.1 to 1, there is a significant shift of the mean landing position of Quasi-Distractor saccades [*t*_(__6__)_ = −2.55, *p* < 0.05] away from the Distractor in the direction of the Target, and a significant shift of Quasi-Target saccades in the direction of the target [*t*_(__6__)_ = −3.62, *p* < 0.05]. Therefore, similarly to valid saccades, invalid saccades tend also to be relatively biased away from the distractor and further toward the salient compound when saliency increases, pointing to the general validity of these effects, regardless of the specific criterion for saccade validity.

**TABLE 1:** Ratios of “Quasi-Target”/“to-Target” and “Quasi-Distractor”/“to- Distractor” saccades.

	SNR 0.1					SNR 1				
Angle	−70°	−45°	0°	+ 45°	+70°	−70°	−45°	0°	+ 45°	+70°
Quasi-Target	12.9%	10.5%	3.3%	7.8%	22.6%	11.9%	6.8%	1.5%	11.0%	15.8%
Quasi-Distractor	26.7%	19.6%	4.3%	18.0%	22.2%	23.5%	17.4%	5.0%	22.5%	23.0%

**FIGURE 11:**
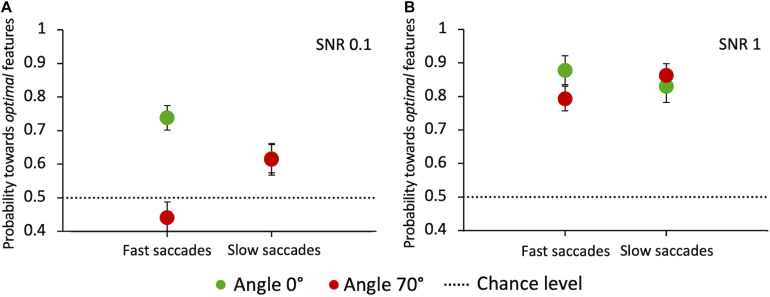
Probability of saccades toward the target as a function of latency, at angles 0° and 70°. **(A)** Probability of saccades toward the target for SNR 0.1. **(B)** Probability of saccades toward the target for SNR 1. Data are pooled across observers (weighted means with errors). “Fast” and “slow” saccades are determined, subject by subject, based on whether they were below or above the individual median latency, respectively. Green circles: angle 0°; red circles: angle 70°. The dashed line indicates the guessing level (0.5 probability).

Finally, we analyzed whether the integration of visual information across time influences the selection of salient features for saccade orientation. If this were true, we would expect the choice performance to vary as a function of saccade latency. A general principle of perceptual decision-making models is that the percentage of correct choices is an increasing function of the response reaction time (Ratcliff and McKoon, 2008). Figure 11 shows, for the two angles that most differ for performance and latency (0° and 70°) and the two extreme SNR values (0.1 and 1), the pooled probability for a saccade to land at the target compound depending on its latency. Latencies were divided into “fast” and “slow” depending on whether they were below or above the individual median latency, respectively. Our results highlight some variability across angles and SNR values. When the target-distractor compound pair is hardly discriminable (SNR = 0.1) and is displayed in the upper hemifield (70° angle), longer-latency saccades lead to a better performance compared to short-latency ones (*Z*-test, *z* = −2.4 *p* = 0.0081). The opposite is true at 0° angle, with a significant decrease of performance for longer-latency saccades (*z* = 2.12, *p* = 0.017), pointing in this case to a disadvantage for target selection performance with prolonged integration of visual information in time. See the “Discussion” section for a possible explanation for this surprising result. With highly salient target compounds (SNR = 1) saccade latency does not have a systematic effect on the choice performance at either 0° or 70° angle, in agreement with the idea that feature-based selection is a fast mechanism that does not benefit from a long temporal integration.

## Discussion

In this work, we found that a specific set of local features, originally identified based on constrained-entropy maximization criteria (Del Viva et al., 2013), are selected as more *salient* than others even in the absence of any global arrangement, both in psychophysical and oculomotor tasks. In past works, the role of those features in early vision had already been shown, but their involvement in saliency determination is evidenced here for the first time.

Psychophysical results show that few *optimal* isolated features are perceived as more salient than the *non-optimal* features by all participants. Their saliency scales with their luminance-contrast and number when presented alone, and with SNR when surrounded by *non-optimal* features. *Optimal* features are so prominent that just one of them can trigger a preferential choice, after having been seen for only 26 ms., both when it is presented alone and when is surrounded by 9 *non-optimal* features. Luminance contrast values are often considered as a reference for saliency comparisons between stimulus dimensions (Nothdurft, 1993a, b, 2000). Ten *optimal* features are still preferred when their luminance-contrast is 65% than that of *non-optimal* features. That is, the saliency instantiated by these particular features, is equivalent to the saliency instantiated by *non-optimal* features with a luminance contrast increased by 35%.

The same pattern of results was obtained in the eye movements experiment. Observer preferentially direct their saccades to the compound including *optimal* features (target), with a probability that increases with the proportion of *optimal* features. We did not find evidence, instead, of a systematic reduction of saccade latency with increasing SNR. This is somewhat unexpected considering that the most widespread models of perceptual decisions assume that response latency is inversely proportional to the rate of accumulation of noisy sensory information (Ratcliff and McKoon, 2008), which in turn is directly proportional to the sensory SNR.

When analyzing saliency discrimination performance as a function of saccadic latency, we observed different effects depending on the angle of presentation of the target-distractor compounds pair and on the SNR. We will come back to the observed anisotropy of oculomotor results later. Overall, under reasonable conditions of visibility (high SNR), postponing the response execution (i.e., increasing the time for integration of sensory evidence) does not seem to help to further improve the selectivity for salient features. Previous studies have reported that short-latency saccades were more strongly affected by salient distractors than slower saccades, suggesting that target selection based on saliency (instantiated by luminance or orientation-contrast) could be facilitated for early saccades (Donk and van Zoest, 2008). A similar fast capture exerted by salient features could explain, in our study, the relative independence of discrimination accuracy upon saccadic latency. More generally, the independence of saccadic latency on SNR is consistent with a fast bottom-up mechanism for saliency extraction, like the one proposed by Del Viva and colleagues (Del Viva et al., 2013), rather than a slower and detailed processing of sensory information.

Saccadic precision instead depends strongly on SNR: the higher the SNR the more precise the saccades directed to the target. Saccades directed to the distractor are instead less precise and further biased in the direction of the target with increasing SNR. This attraction bias toward the salient compound is independent on the validity criterion of saccades chosen in this study.

All together, these results point to a rapid orientation of saccades toward the salient information provided by *optimal* features.

Humans can only fixate and extract detailed information from one small region of space at a time. This makes an efficient selection of relevant local features critical for visual processing and optimal behavior. Decades of work in vision science have argued for such dynamic selection to be based on multiple saliency maps (Itti et al., 1998; Itti and Koch, 2001; Parkhurst et al., 2002; Torralba, 2003). The saliency of *optimal* features is independent of the global image context, leading to speculate that they may play an important role within the multi-scale analysis of saliency performed by the human visual system.

The saliency map is not derived, in our case, from an algorithm trying to make sense of visual properties determined *a priori* (e.g., color, motion, texture) competing at individual image locations. Our salient features are instead a consequence of both the early input data reduction, needed by the visual system due to its limited processing capacity for the costs of neural activity and structural limits (Attwell and Laughlin, 2001; Lennie, 2003; Echeverri, 2006), and of the frequency with which they occur in the input. A few of these distinctive features are in fact more significant than others, despite having similar low-level properties (luminance, spatial frequency, size), because they represent a compromise between the information they carry about the visual scene and the cost for the system to process them. On the other hand, the alternative *non-optimal* features used in the present study are individually the most informative, but do not meet computational limitations criteria (Del Viva et al., 2013). Therefore, the computational limitations do much more than simply limit the performance of the system, they seem to take a significant role, not only in compression, but also in shaping what the system selects as salient in the input.

Several past studies have explored the mechanisms of fast vision at different scales and stimulus durations, finding that both coarse and fine spatial information are simultaneously used in fast categorization of images (Oliva and Schyns, 1997; Schyns and Oliva, 1999). Some models build bottom-up saliency maps, based on simultaneous processing of different visual properties at multiple spatial scales that are then somehow combined into a single saliency-map (Itti and Koch, 2001). These models do not address the issue of the amount of computing power required by each of these parallel processes that varies greatly across scales and modalities. Our model instead revolves entirely around the concept of computational costs. From this viewpoint, the finest usable spatial scale takes naturally a central role. As a consequence of the properties of the Fourier transform, the information content is proportional to the square of spatial frequency, making the finest scale by large the most computationally demanding part of the processing. As a consequence, saliency extraction at this scale, with strong reduction of information, becomes a pressing necessity, and one expects it to play an important role amongst all possible maps involved.

There is still a debate on whether this fast bottom-up extraction of visual saliency map is based mainly on local (Li, 2002) or rather global clues (Oliva and Schyns, 1997; Itti et al., 1998; Torralba, 2003; Oliva, 2005). The contribution of local analysis to the global percept of an image has been studied in a past work, within the framework of the present model, by replacing in a sketch the *optimal* features (typically located along within objects contours), with other features that are *non-optimal* carriers of information, keeping their localization in the image unchanged. The disruption of these *optimal* local cues causes a decrease of image recognizability, in spite of its global structure being preserved (Del Viva et al., 2016). While the existence of other mechanisms in addition to what is analyzed here has been proved beyond doubt, this new result allows to establish the existence of a bottom-up reference frame for the extraction of saliency that can efficiently drive the process.

Many studies have proposed that bottom-up saliency maps are represented in early sensory cortices (Zhaoping, 2006, 2019; Zhaoping and Zhe, 2015) and rely on specific sensory properties. Priority maps are instead less dependent on the detailed physical properties of the sensory input, and account for both the global properties of the scene, the behavioral goals and high cognitive information (as reviewed in Itti and Koch, 2001; Zelinsky and Bisley, 2015). They would rather be represented in higher cortical sensory areas (including parietal and prefrontal areas, Thompson and Bichot, 2005; Bisley and Goldberg, 2010), as well as in subcortical regions closer to the motor output, as the Superior Colliculus for saccade planning (Veale et al., 2017; White et al., 2017). Both earlier studies, supporting the view of saliency maps as represented in early visual cortex (Zhaoping, 2006), and more recent works, suggesting the existence of a priority map in the superior colliculus (Veale et al., 2017; White et al., 2017), agree on the fast nature of such representations. The gaze could then be rapidly oriented toward the maximum-saliency locations highlighted by these maps (Itti and Koch, 2000, 2001; Najemnik and Geisler, 2005, 2008; Itti and Baldi, 2009; Garcia-Diaz et al., 2012).

The saliency map extracted by the constrained maximum entropy algorithm, for efficient compression, must be created very early in the visual system, and several converging evidence indicate as the most likely candidate the primary visual cortex. First of all, *optimal* features are good approximations, within the limitations of a 3 × 3 grid, of the structure of some receptive fields of neurons found in primary visual areas (Hubel and Wiesel, 1965). Such elongated edge- and bar-shaped structures haven’t been found in the thalamus and superior colliculus (Harutiunian Kozak et al., 1973; Drager and Hubel, 1975; DeAngelis et al., 1995; Kara et al., 2002; Derrington and Webb, 2004), although some studies found orientation selectivity in the superior colliculus (Wang et al., 2010; Gale and Murphy, 2014; Ahmadlou and Heimel, 2015; Feinberg and Meister, 2015; De Franceschi and Solomon, 2018). Then *optimal* features extraction supports a fine-scale local analysis consistent with V1 (Hubel and Wiesel, 1962, 1974; Lennie, 1998). V1 is also the most extended visual area (Lennie, 1998), with larger energy consumption (Lennie, 2003) and higher input/output neural ratio with respect to the retina and other extrastriate areas (Lennie, 1998), making it a good candidate for the information bottleneck required by our model. Finally, V1 is involved in very fast visual analysis (Grill-Spector et al., 2000; Kirchner and Thorpe, 2006). All these observations are consistent with the idea, previously advanced, that the function of V1 is to create a “bottom-up saliency map” enabling a “lossy pre-attentive selection of information,” so that data rate can be further reduced for detailed processing (Zhaoping, 2006; Zhaoping and May, 2007; Zhaoping, 2019).

The visual system is capable of detecting very quickly potentially dangerous or very interesting stimuli to activate emotive or fight-or-flight autonomic responses essential for survival (Morris et al., 1999). This analysis does not need, and probably does not use, detailed visual information but needs fast and reliable processing of relevant elements (LeDoux, 1996; Öhman et al., 2001; Perrinet and Bednar, 2015). This processing could take advantage of a quick inspection of different small regions distributed over the image, each providing enough information about the whole scene. For this reason, it could use a constrained maximum-entropy approach to extract a saliency map, that the oculomotor system could use to drive eye movements toward potentially relevant locations (Itti and Koch, 2000, 2001; Najemnik and Geisler, 2005, 2008; Itti and Baldi, 2009; Garcia-Diaz et al., 2012; Schütz et al., 2012).

Such rapid and optimal selection of information, devoid of detailed fine-scale color or luminance information (Del Viva et al., 2016), could be sufficient *per se* to provide salient locations in first viewed scenes that could be followed, only at those locations, by a more detailed analysis. This would require a much larger computational power and may be only possible if performed more slowly and/or on a reduced part of the image. Our hypothesis does not exclude other rapid simultaneous processing of large-scale visual properties, that do not need such compression (e.g., Gegenfurtner and Rieger, 2000).

As an aside, a clear up-down anisotropy has been found in oculomotor data, that is arguably connected with stimulus saliency, but still deserves a brief discussion and future investigations. Saccades oriented to the upper visual field had a dramatically reduced latency with respect to the lower visual field, even more pronounced than in previous studies (e.g., Honda and Findlay, 1992). The increased latency for horizontal compared to upward vertical saccades found here might be due to the bilateral presentation of the target-distractor pair, which is known to maximize the Remote Distractor Effect on saccades latency (Walker et al., 1997; Benson, 2008). These phenomena have been attributed to purely oculomotor properties, rather than to visual processing mechanisms (Honda and Findlay, 1992; Walker et al., 1997), coherently with the relative independence, in our data, of the latency on SNR. Finally, the lower performance in the upper hemifield probably reflects the superiority of perceptual discrimination in the lower visual field (see for example Talgar and Carrasco, 2002).

To conclude, the results presented in this paper suggest that saliency may be derived naturally in a system that, under the pressure of fast visual analysis, operates maximum information transmission under computational limitation constraints. They also suggest that these salient features participate early in the visual reconstruction process that must be, at least partly, initiated at the local level. We also speculate that active vision is efficiently adapted to maximize information in natural visual scenes under specific processing constraints.

Since our attention automatically shifts to salient targets (Nothdurft, 2002; Theeuwes, 2010), one of the challenges for future research will be to investigate whether *optimal* features can rapidly and automatically attract the subject’s attention more than others in covert and overt (oculomotor) tasks, in which “saliency” is implicitly manipulated rather than explicitly cued, as in the present work. It would be also interesting to assess the strength of automatic attention capturing of these local isolated features compared to global visual elements.

## Data Availability Statement

The datasets presented in this study can be found in online repositories. The names of the repository/repositories and accession number(s) can be found below: Zenodo (doi: 10.5281/zenodo.4620864).

## Ethics Statement

The studies involving human participants were reviewed and approved by the Comité d’éthique d’Aix-Marseille Université, ref: 2014-12-3-05 and Comitato Etico Pediatrico Regionale—Azienda Ospedaliero-Universitaria Meyer—Firenze FI. The patients/participants provided their written informed consent to participate in this study.

## Author Contributions

SC participated in the data collection, statistical analysis, and manuscript writing. AM and MD participated in the project ideation, experiment programming, statistical analysis, and manuscript writing. All authors contributed to the article and approved the submitted version.

## Conflict of Interest

The authors declare that the research was conducted in the absence of any commercial or financial relationships that could be construed as a potential conflict of interest.
